# Field Test of Self-Cleaning Zr-Modified-TiO_2_-SiO_2_ Films on Glass with a Demonstration of Their Anti-Fogging Effect

**DOI:** 10.3390/ma12132196

**Published:** 2019-07-08

**Authors:** Andraž Šuligoj, Olena Pliekhova, Nives Vodišek, Mohor Mihelčič, Angelja K. Surca, Roman Kunič, Barbara Šubic, Jernej Starman, Aleš Ugovšek, Urška Lavrenčič Štangar

**Affiliations:** 1 Faculty of Chemistry and Chemical Technology, University of Ljubljana, Večna pot 113, SI-1000 Ljubljana, Slovenia; 2 Department of Inorganic Chemistry and Technology, National Institute of Chemistry, Hajdrihova 19, SI-1000 Ljubljana, Slovenia; 3 University of Nova Gorica, Vipavska 13, SI-5000 Nova Gorica, Slovenia; 4 Faculty of Civil and Geodetic Engineering, University of Ljubljana, Jamova 2, 1000 Ljubljana, Slovenia; 5 M-Sora d.d., Industrijska ul. 13, SI-4226 Žiri, Slovenia

**Keywords:** self-cleaning, anti-fogging, TiO_2_-SiO_2_, field test, glazing

## Abstract

The number of commercial products claiming self-cleaning properties is rising and testing of long-term activity and durability of such coatings needs to be addressed more. The time-dependent changes of different characteristics like haze, transparency, and color are essential for transparent glazing materials. Herein, we aimed to examine whether the laboratory results obtained on the Zr-modified-titania-silica (TiZr) self-cleaning materials would translate to larger-scale outdoor-exposed testing. TiZr thin films were deposited via spraying onto float glass window surfaces and exposed into three different environments for 20 months. For comparison, a commercially available active SGG BIOCLEAN^TM^ glass and standard float glass were simultaneously exposed in the same conditions. It was shown that the self-cleaning property of either a commercial product or TiZr-coated float glass was not considerably effective in real field test conditions, although the previous laboratory tests showed pronounced photocatalytic activity of TiZr thin films. The inclination angle; however, was shown to have a considerable effect on the self-cleaning ability of samples, as did the rain patterns during the testing period. On the other hand, the anti-fogging effect of our TiZr material was very well expressed in controlled laboratory conditions (measuring droplet formation time) as well as in the real outdoor environment.

## 1. Introduction

Dust and pollution deposition on the operational surfaces of glass, concrete, metallic, textile, and plastic items is an actual problem, thus creating scientific and industrial interest in self-cleaning and self-sterilizing coatings [1,2]. Easy-to-clean surfaces with minimum human intervention significantly reduce maintenance costs and are one of the prerequisites to modern operational surfaces fabrication. The number of commercial products claiming self-cleaning, hydrophobic, or hydrophilic properties have currently reached a couple of dozens [3] and testing of long-term activity and durability of such coatings needs to be addressed more.

Glass is an extremely important and useful material due to its transparency and is used for fenestration, automobile windshields, and display production and even as a construction material. More recently, demand and number of transparent glazing coatings for solar photovoltaic (PV) panels have increased as well. The transparent coatings with different functionalities are in great demand for glass materials. The early publications claimed a self-cleaning effect of the coated glass even when it was not exposed to water [4]; however, another study [5] clearly showed, that additional service-like rinsing of self-cleaning glass would be necessary anyway.

Keeping transparency and avoidance of fouling during the glass surfaces usage is very important for all applications mentioned. However, the dust is not the only problem. An additional problem is the condensation of moisture, which condenses onto a cold surface of the glass. Theoretical calculations showed that the condensation on glass surfaces could be prevented up to a certain degree and special glazing with anti-fogging coatings could additionally help in that process [6].

A common component of highly transparent self-cleaning and anti-fogging glazing is silica coupled with a photocatalytic material, for example, TiO_2_ [7]. SiO_2_ is a material with a low refractive index and possesses many advantages such as chemical resistivity, optical transmittance, high morphological diversity, and anti-reflective properties due to the mesoporous structure of the final film. In addition, it can be effectively used as a binder to increase the coating mechanical resistivity.

Titanium dioxide (TiO_2_) has been established as a highly active photocatalytic material in various applications and has received much attention in the scientific community over the last two decades [8,9]. However, the high refractive index of TiO_2_ (2.52 for anatase) makes it challenging to introduce the required amount of TiO_2_ into the coating to ensure its self-cleaning performance while still maintaining the transparency of the coating (i.e., keeping the refractive index between 1.21 and 1.23). Increasing the porosity of the coating (including more void, i.e., air, volume), one can reduce the effect of the high refractive index of TiO_2_; however, this may lead to mechanically fragile films [10]. The film thickness affects transparency as well [11]. In general, a limited amount of TiO_2_ particles can be dispersed in the coating. It was demonstrated that the inclusion of SiO2 to porous TiO2 films allows for correction and adjustment of structural colors of the films [12]. Pinho and Mosquera have deposited TiO_2_-SiO_2_ coatings on stone for its protection [13]. They have found that larger and sharper titania particles were beneficial in catalytic activity, mainly due to increased availability of the active sites on titania. This; however, results in lowering the transmission of the layer, which is important in self-cleaning coatings on windows, hence smaller nanoparticles are preferred in these applications.

Our previous research has shown that applying a self-cleaning TiO_2_-SiO_2_ thin film did not decrease the absorption of incident light for photovoltaic applications even after 2 months of exposure [2]. Moreover, SiO_2_ protective layer was shown to prevent sodium diffusion to TiO_2_ phase [14], while an effect of protecting the otherwise photo-labile polymer substrate was shown with TiO_2_-SiO_2_ composite films [15]. All this modulation and versatility is commonly reached with the aid of sol-gel chemistry as a synthesis procedure.

Sol-gel has been successfully applied for silica, titania, or composite (hybrid) glazing fabrication [16]. It is an easy and convenient process employing mild conditions and at the same time allowing for the preparation of homogeneous composites with unique and controllable properties. For example, in TiO_2_ synthesis, the technique allows for the formation of anatase phase even at low temperatures [17], especially when titanium tetraisopropoxide is used as the alkoxide and acid acts as an electrostatic stabilizer and a hydrolysis catalyst [18]. Combination of titania and silica has been studied also in the light of the effects on photocatalytic activity. Hakki and co-workers [19] postulated that the greater the amount of Ti-O-Si bonds in the binary oxide system, the lower the nitrate selectivity towards NO_X_ abatement. However, this resulted in stronger coatings, which were better adhered to the surface. Thus, produced sols also enable deposition using spraying, which is highly important in the case of larger surfaces, as needed for the in-field exposures, because although dip-coating and doctor-blade techniques allow for more control in the industrial production of the glazings, these methods are not applicable onto already-installed glass surfaces.

Different testing approaches can be used for the evaluation of the quality of coatings during in-field exposures, such as measurements of haze, color change, and light-transmittance. Each of them has its own advantages and drawbacks but together they can offer important information on the behavior of long-term exposed surfaces. For example, haze values give the scattering of light by a plane-parallel surface. The method is recommended in the documentary standard ASTM D1003 [20]. The scattered light is responsible for the reduction in the contrast of the objects that are viewed through the investigated glazings.

Another important characteristic of the functional coatings is color. Color differences can be described as the relative distance between two mathematically specified colors, according to the International Commission on Illumination (CIE) [21]. The difference is usually shown as ΔE and is calculated by comparing reference and sample L*a*b* values. The drawback of ΔE data is that calculations quantify the magnitude of a color difference but do not necessarily indicate the direction of the difference. However, portable instruments are available, which enables in-field measurements.

The change in transparency of the long-term exposed objects is another important characteristic for initially transparent materials like glass, coatings. It can be measured through the ratio in the intensity of light transmitted by the glazing and intensity of the light falling on a surface. Transparency is easily definable with a lux meter by measuring material total transmittance and depends on the absorption and reflection factors [22].

Our previous studies showed that sol-gel-derived titania modified with Zr, which is trapped into a silica matrix, results in a photocatalytically-active TiZr-SiO_2_ thin film, suitable for application onto various surfaces [15,23]. Herein, we aimed to expose the up-scaled laboratory samples (i.e., TiZr-SiO_2_ self-cleaning materials) on float glass window surfaces to different environments, ranging from an urban area with industrial complexes to a more remote and rural-type area. In addition, we installed coated glass samples with a different tilt to the horizon (45°, 90°, and covered/under the roof at 90°). For comparison, two commercially available surfaces (i.e. uncoated float glass and active SGG BIOCLEAN^TM^ surface) were simultaneously exposed. It was demonstrated earlier that the reduction in transmittance of glass is closely related to the density of dust deposition and is a function of the tilt angle [24]. The transmittance, CIE L*a*b* and haze characterizations of the exposed surfaces were made at regular intervals. A 20-month exposure period with different window configurations enabled us to study the parameters influencing the stability and self-cleaning activity of such coatings. A discussion with respect to the parameters that affect the self-cleaning performances of different surfaces is provided. Moreover, the anti-fogging ability of the TiZr surface was evaluated through the determination of the droplet formation time in the laboratory, as well as on the outside surface of a real built-in window.

## 2. Materials and Methods

### 2.1. Preparation of Materials

The TiO_2_ sol was prepared through sol-gel reflux processing, as previously described [23]. Briefly, 15 mL of titanium tetraisopropoxide (TTIP) and 2.5 mL of absolute ethanol (EtOH) were mixed in a flask. Depending on the pre-determined Zr/Ti ratio, zirconium tetrabutoxide (ZTB) was added (0, 5, 10, and 20 mol.%) and the mixture was stirred for 90 min. Separately, 45 mL of deionized water and 1 mL of perchloric acid were mixed. This solution was then dropwise added to the alkoxide solution, which resulted in hydrolysis and condensation exothermic reactions of metal alkoxides, giving a white precipitate of hydrated amorphous TiO_2_ and ZrO_2_. SiO_2_ binder solution was prepared from tetraethyl orthosilicate (98%), colloidal SiO_2_ (Levasil 200/30% by Obermeier, Bad Berleburg, Germany), HCl, and 2-propanol. TiO_2_ and SiO_2_ sols were combined and mixed thoroughly overnight. A mixture of 1-propanol and 2-propoxyethanol were finally added to produce a solution with suitable rheological properties [23]. This sol was then deposited on float glass using an airbrush (DS-32, 0.35 mm nozzle diameter, Fimotool, Slovenia) with the air-flow of 15 L/min and 2.5 bar of pressure. Spraying was made from a distance of 15 cm in circular moves three times in order to achieve sufficient coverage. In between, the layers were dried with an air heat gun (Black & Decker, Towson, MD, USA) operating at 70 °C. Note that although the final solution was a mixture of titania (modified with 10% Zr) and silica, for the sake of simplicity, such coatings were labeled as TiZr.

For field tests, double-glass wooden windows (30 × 30 cm^2^) were built. Each window consisted of back float glass and the front (exposed) glass. For float and SGG BIOCLEAN^®^ (abbreviated BioClean, Courbevoie, France) front glass specimens the as-supplied glass panels were installed, while for TiZr surfaces, float glass on the front side was coated with a sol of Zr-modified TiO_2_-SiO_2_, as described above.

### 2.2. Field Tests

Three types of window surfaces (two equal specimens for each) were exposed in long-term (20 months) field experiment in three different areas in Slovenia. The areas of exposure represent:

- Ljubljana (abbreviated LJ): large city in the urban area with industrial complexes, moderate continental climate;
- Nova Gorica (abbreviated NG): city in the urban area, moderate Mediterranean climate;
- Žiri (abbreviated ZI): a small industrial complex in the rural and remote area, moderate continental climate.

The samples were exposed at three different tilt conditions (i.e., 45°, 90° and at 90° but sheltered by the roof; Figure S1 in Supplementary Materials).

For haze measurements, the same three kinds of surfaces were exposed in the field. However, the dimension of these samples was 4 × 9 cm^2^ with the aim to be appropriate for the laboratory spectroscopic measurements.

### 2.3. Characterization

Transmittance was measured by using a handheld lux meter (LED light meter) (Extech Instruments, Nashua, NH, USA). Two lux meters were positioned on a levelled surface, one lying outside/beside and one under (through) the window (Figure S2 in Supplementary Materials). The difference between the two lux meters positioned beside each other (outside the window) did not exceed 0.5%. The results were calculated as

R = Θ_v_/Θ_Vglass_, (1)

where Θ_V_ and Θ_Vglass_ are the fluxes outside/beside and under the window, respectively. For easier interpretation, a deviation from an ideal transmittance is given in the plots as 1-R. In this case, values are increasing with time, as the windows get less transparent due to dirt deposits. A lux meter measures luminous flux, which is scaled to reflect «visible flux» by using the luminosity function. The results were averaged from three different measurement positions below the tested sample (left, middle, right position on the opened edge of the window sample).

CIE L*a*b* characterization was used as a way of assessing the change of color (ΔE) by using a handheld meter (EasyCo, Erichsen, Germany). The apparatus was measuring the L*, a*, and b* coordinates of the glass with a whiteboard positioned under it. The whiteboard was taken as a background and, since the technique enables differentiation between the contributions from the light scattering expressed by ΔL* and the color changes described by Δa* and Δb* values, the global change of color (ΔE*) values were obtained from the equation

ΔE_ab_* = (ΔL*^2^ × Δa*^2^ × Δb*^2^)^1/2^_._ (2)

The difference (Δ) was measured as the difference of the parameters (L*, a*, b*) measured with glass underneath from those of the whiteboard (see Figure S3 in Supplementary Materials). Each measurement was averaged over five data points at different positions (four in the corners of the window and one in the center).

The laboratory measurement of the haze was performed on small samples (4 × 9 cm^2^) after in-field/laboratory transfer for each measurement. Haze value is according to ASTM D1003 defined as the ratio of diffuse to total luminous transmittance (DT/TT %). Luminous transmittance is weighted with regard to the relative sensitivity of the human eye in the photopic state. DT and TT were measured on a Perkin Elmer Lambda 900 UV-VIS spectrometer (Waltham, MA, USA) that is equipped with an integrating sphere. Both transmittances were measured in the 380–780 nm range using 10° CIE standard observer and CIE standard illuminant A. Transmittance spectra were recorded at 23 ± 2 °C.

X-ray powder diffraction (XRD) patterns were measured on a RIGAKU MiniFlex 600 (Rigaku, Tokyo, Japan) apparatus with the copper source, providing X-rays of the wavelength of 0.154 nm. The samples were scratched from the coated glass and ground in agate mortar prior to analysis.

Atomic force microscopy (AFM) images were recorded on an AFM attachment of WITec alpha 300 confocal Raman spectrometer (Ulm, Germany). The images were recorded on areas of 20 × 20 mm^2^. Scanning electron microscopy (SEM) was done on a Zeiss Supra 3 VP field emission gun (FEG) microscope (Jena, Germany) operating at 2.0 kV.

The condensation experiment was conducted in a laboratory environment at 22 °C at three different relative humidities (44%, 52%, and 58%). An aluminum block (20 × 20 × 8 cm^3^) was put in the freezer (−12 °C) to cool. The block was put out and three samples of glass/coatings were placed on its surface in a horizontal and vertical position. The time of the visual appearance of condensate water droplets was measured.

Water contact angles were measured on a Theta Lite goniometer (Biolin Scientific, Västra Frölunda, Sweden) using Milli-Q water. The samples were pre-cleaned under UV-illumination (365 nm, 45 W) for 12 h and then stored in dark for approximately 1 h before measurement. Each measurement consisted of five points on a 20 × 20 mm^2^ sample size.

## 3. Results and Discussion

### 3.1. Coatings Characterization

**Figure 1:**
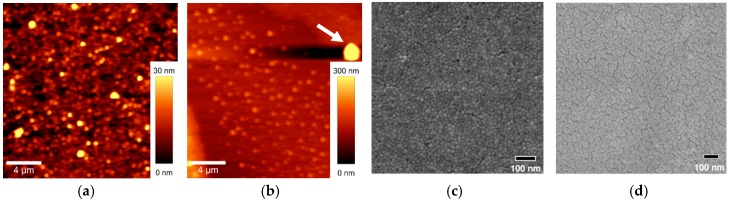
Atomic force microscopy (AFM) (**a**,**b**) and scanning electron microscopy (SEM) (**c**,**d**) images of the TiZr coating obtained with the dip-coating (**a**,**c**) and spray-coating method (**b**,**d**).

Our previous reports discussed in the sections above were dedicated to investigating TiZr coatings prepared by dip-coating technique with different Zr contents. The best compromise between photocatalytic activity and mechanical stability was achieved with coatings comprised of 10% Zr. Hence this coating was used in this in-field study and is labeled TiZr throughout this study. However, as stated in the introduction, for large in-field surfaces the use of dip-coating is not appropriate and effective; spray-coating deposition is preferred but can influence the surface characteristics. Therefore, surface roughness and morphology of TiZr films were analyzed with AFM and SEM microscopy (Figure 1) not only for dip-coated but also for spray-coated samples—which were eventually used in the study. The measurements revealed differences between surfaces obtained with the two deposition methods. On the AFM images, dip-coated films show a nanocrystalline structure with the surface roughness of 39 nm. This value significantly increased (to 256 nm) for spray-coated films. This surface revealed the presence of more separated agglomerates, as marked with an arrow in Figure 1b. Importantly, most AFM images were characterized by at least one such large particle with a dimension of up to 1.5 μm, which surely raised the calculated surface morphology significantly. Obviously, the forces of the air flow during the spraying to some extent reinforce agglomeration of the TiO_2_ particles. Petronella et al. have discussed the degree of aggregation in spray-coated TiO_2_ films [25]. They have concluded that the use of an appropriate solvent has a great effect on aggregation degree due to capillary forces during the solvent evaporation on the coated surface.

SEM images show similar morphology as observed with AFM technique; the dip-coated films have a slightly more porous structure and spray-coated glazings show a uniform surface with the scarce presence of slightly larger aggregates (not shown in Figure 1d). Hence, the spray-coated technique, although resulting in slightly larger aggregates, offered adequate coating quality and was used in the field tests.

**Figure 2:**
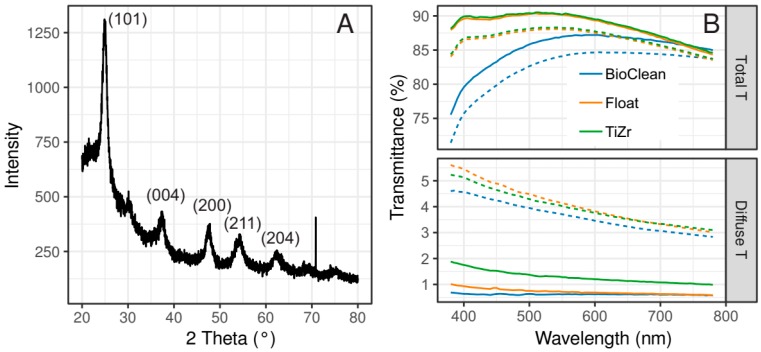
X-ray diffraction (XRD) pattern of dried TiZr sol (**A**) and UV-vis total (Total T) and diffuse transmittance (Diffuse T) of the three tested surfaces (**B**). The solid (—) and dashed (---) lines in (**B**) represent fresh and used (20 months of exposure) 90° samples averaged across all three locations, respectively.

Figure 2A represents the characterization of TiZr composite with X-ray diffraction technique. There, the proof of the presence of anatase phase of titania is shown. The reflections of (101), (004), (200) and other planes are clearly showing adequate crystallinity. The presence of SiO_2_ phase in the TiZr composite coatings is not seen due to its amorphous character and disordered structure. Its porous structure, on the other hand, is indicated by the anti-reflective property as shown in total transmittance measurements (Figure 2B), where the addition of the coating slightly increased the transparency in the visible part of the spectrum. Hedayati and Elbahri [26] have summed the different mechanisms for anti-reflective effects and considering two- or three-layer coatings—as is in this case—benefit the most from the decreased refractive index by the introduction of the porous structure of the coating. In contrast, the total transmittance of the BioClean surface is considerably lower at wavelengths up to 700 nm. This coating is also characterized by a light blue shade. Diffuse transmittance, on the other hand, is the highest for the TiZr coating (1%–2%), which reflects its nanocrystalline structure. The values are more similar for float glass and BioClean surfaces, both being below 1%. Such values are low enough to give firm evidence of the transparency and uniformity of the glass/coatings. The curves of samples at 90° inclination (averaged across all three locations) after 20 months of exposure are shown as dashed lines (Figure 2B). As expected, the total transmittance of all shifted curves was reduced while diffuse transmittance increased; the haze data, obtained from these curves, is discussed in more detail in the following section.

### 3.2. Field Test Results

**Figure 3:**
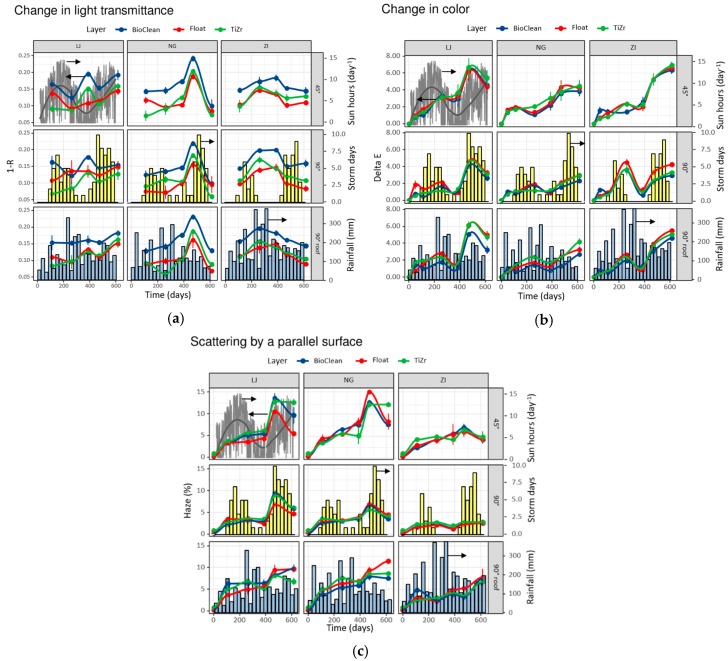
Results of field testing of the surfaces; change in light transmittance is expressed as change in transmittance (1-R) according to lux meter (**a**), color changes (ΔE*) (**b**), and changes in haze (**c**). The blue and yellow columns represent the average monthly rainfall and the number of days with storms in one month on the three locations, respectively, and are valid for all panels. The gray lines in the top-left panel represent the number of hours of sun per day and are valid for this location only (Ljubljana). Results are faceted by areas (vertical facets) and angles of exposure (horizontal facets).

The results of transmittance, color change ΔE*, and haze are shown in Figure 3 and are faceted by areas (LJ, NG, ZI—vertical facets) and angles of exposure (45°, 90°, 90° roof—horizontal facets). Each sample window is presented with a certain curve color (blue—BioClean commercial glass pane, Courbevoie, France; red—float glass pane; green—experimentally prepared float glass with TiZr thin film). Since rainfall can considerably contribute to the results, patterns for all the three areas are depicted as the blue columns, which represent the average monthly rainfall; and yellow bars, which represent the number of days having a storm. They refer to all inclinations although they are shown only in one panel, for clarity. The data for sunny hours per day was accessible only for one location (Ljubljana, Slovenia), hence it is depicted as the gray curve in the top left panel.

Three different techniques were used to characterize the samples during the exposure, and because they represent different aspects of the tested materials, we first discuss the results of each of them and only later discuss the more holistic conclusions. The temporal trends as measured with lux meter are shown in Figure 3a as a portion of the light that does not pass through the window. In other words, the higher this portion, the lower the transmittance of the light as detected by the human eye. For this technique the results show:

- Similar trends for float and TiZr specimen;
- BioClean reveals lower transmittance values (higher 1-R) due to a light bluish tint of this glass;
- The smallest variations are found for Žiri (ZI) location (rural and remote area);
- The inlet of Saharan sand at ca. 480th day was significantly contributing to obstruction of transmitted light, the largest at Nova Gorica (NG) location with Mediterranean climate;
- No correlation could be seen with solar irradiance (number of sun hours per day) in any of the inclinations;
- Angles of exposure did not influence the measured parameters significantly.

Figure 3b shows the color change ΔE* through CIE L*a*b* determination during exposure of window specimens:

- The temporal changes for all types of samples overlap significantly;
- ΔE* values generally increase with time of exposure;
- The inlet of Saharan sand contributed to a more expressed increase of ΔE* at all three locations;
- Solar irradiance correlated with bigger changes in ΔE* values;
- Differences among angles of exposure are well seen for ΔE* measurements—the samples exposed at 45° and 90°-roof exhibit the highest increase in ΔE*.

Similar trends to ΔE* measurements are also observed in the case of haze determination (Figure 3c):

- The curves for all types of samples overlap significantly;
- Haze increases with time of exposure, the lowest values were found for the remote area of ZI (the samples remain cleaner—forestry area around);
- The inlet of Saharan sand contributed to a more expressed increase of haze at NG and Ljubljana (LJ), much less for location in ZI;
- Solar irradiance correlated with bigger changes in haze values;
- Differences among angles of exposure confirm that the rain flow cleans the surface dirt more efficiently when exposed at 90°.

**Figure 4:**
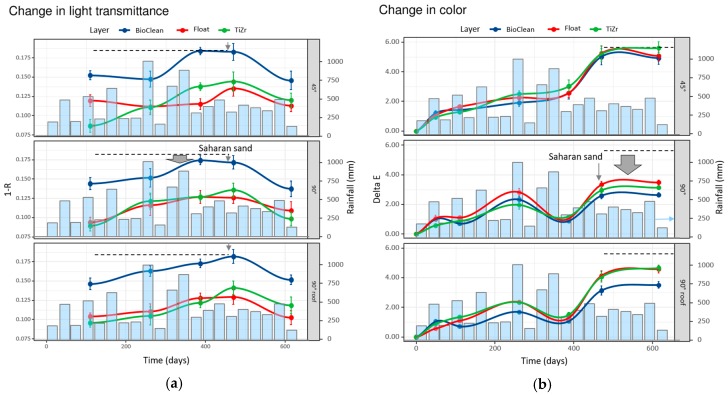

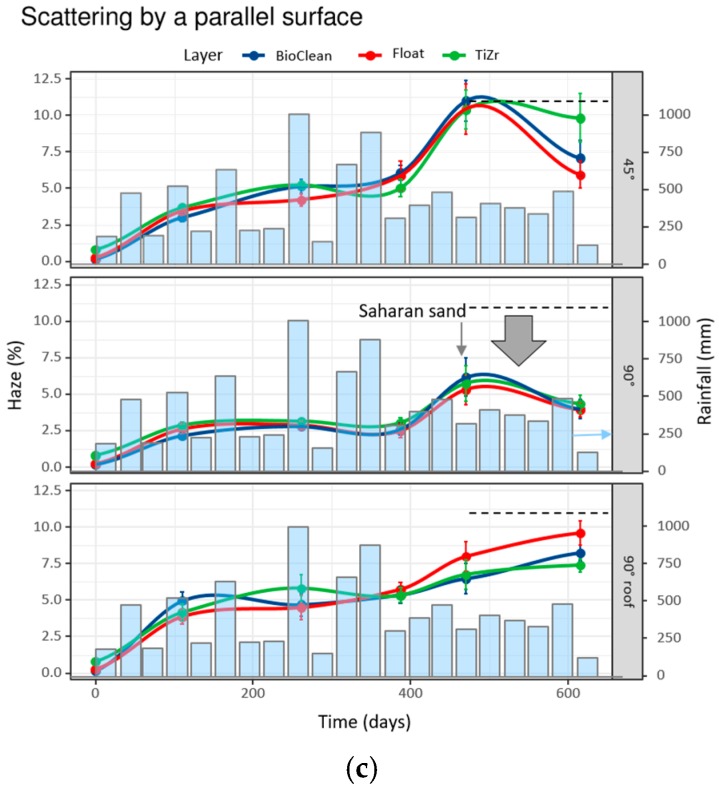
The averaged results from the three locations of field testing of the surfaces; change in light transmittance expressed as 1-R according to lux meter (**a**), changes in ΔE* values; (**b**) and changes in haze (**c**). The pale blue bars represent the average monthly rainfall in mm across all locations. The dashed lines represent the maximum value as measured in the dataset, and are shown to guide the reader’s eye, and the gray-filled arrow represents the difference from the maximum values (measured at 45° inclination).

From measurement results in Figure 3, some trends that directly correspond to the weathering conditions, locations, and inclinations of the sample can be observed. However, due to a large number of samples, certain deviations were noted. Since each testing method has its own advantages and drawbacks, and, additionally, different measurement points on the sample contributed to the scattering of results, the averaged results from the three locations are shown in Figure 4.

In general, the orientation of the windows that provided the highest transmittance and, hence, self-cleaning effect was 90° and the inclination with the most accumulated dirt was 45°, as shown with the gray-filled arrows showing the difference in values among 45° and 90°.

**Figure 5:**
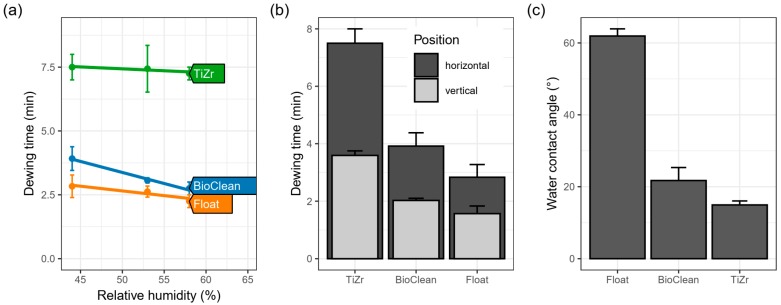
Dependence of time of formation of water droplets (condensation) on the relative humidity across samples (**a**), dependence on the orientation of the sample (**b**) and water contact angles of the pristine samples (**c**). Solid lines in (**a**) represent the linear correlation fitting to data.

As already expressed above for different locations (Figure 3b,c), the time trends in ΔE* (Figure 4b) and haze (Figure 4c) values are practically identical. Although it is difficult to say precisely since many factors (such as surface structure or observation conditions) affect the human perception of color differences, the limit of perceptibility is usually taken in the range of 2–4 CIELab units [27,28,29]. As seen in Figure 1b, only the 90° inclination succeeded to stay below the 4 CIELab unit differences, which implies the importance of surface water runoff for self-cleaning effect. The most noticeable decrease in quality of coatings/glass happened after circa 480 days of exposure, which coincides with the appearance of Saharan sand in the atmosphere in the area at that time (see Figure S4 in Supplementary Materials for meteorological evidence of the event, up to 1000 mg/m^2^ of sand deposit). The increase in ΔE* and haze was ca. 2.8- and 2-fold, respectively, and the same phenomenon was noticed with lux meter measurements (Figure 4a). After that, the coatings seem to recover, become cleaner again and the trends were leveled. That being said, not all windows recovered at the same pace. Most notably, in the 45° inclination, TiZr coating consistently performed the worst—the least so with the lux meter technique. As proven later (see Figure 5), the TiZr surface exhibited the highest hydrophilic nature, and this effect was more expressed in the horizontal position. It seems the combination of inorganic (non-degradable) debris on the coating’s surface and low inclination angle (45°) prevents the formation of a large-surface thin layer of water, which is formed with super-hydrophilic surfaces. This results in lower runoff and consequently in less cleaning effect.

As with the lux meter, the biggest factor influencing the behavior of surfaces was the position of the windows. The biggest changes were detected in windows with 45° installation, while the smallest in 90° inclination; the 90° installation with a roof performed a little worse in all cases. This can be explained with the washing effect, where the water from rain cleans 90° surface more efficiently than 45° due to greater gravitational power, while the 90° surface under the roof receives less rain in general. The same effect is connected also with Saharan sand (ca. 480 days of exposure, see Figure S4 in Supplementary Materials), which most affected the surfaces positioned at 45° angle (highest surface for vertical settling), while both other positions received less impact. Sakhuja et al. [30] have also studied the effects of inclination on self-cleaning hydrophilic nanostructured glass and concluded that in order for the self-cleaning effect to take place a certain inclination is mandatory—their tilt was set at 20°. Gholami et al. [31] on the other hand, studied the same parameter and concluded that the addition of hydrophilic TiO_2_ coating was effective against dust accumulation even in periods without rain. It must be noted that both of the above studies were relatively short term (five and ten weeks), hence longer studies like this one could come to different conclusions. Especially interesting is the influence of sunlight exposure (Figure 3). Looking at the 90° inclination, especially for ΔE* and haze measurements, the rate of increase in these two values was relatively low in the time of high solar exposure; it increased dramatically at exposure day 480 and only started to decrease after high rain period in combination with high sun exposure. This implies the combined beneficial effect of solar exposure and rain.

All three measurement techniques confirmed the trend after pollution with inorganic sand (day 480), that is, they all show general improvement of the surfaces with the period of steady rain which followed this event, showing the importance of exposure of surfaces to rainwater. What is more, apart from lux meter measurements, surfaces receiving high amounts of strong rain (storm days as yellow bars in Figure 3) recovered from the Saharan sand the fastest. This can be seen in the location of Ljubljana, which had the highest number of storm days following the sand deposit and, consequently, cleaned the surfaces the fastest and to the highest degree. Despite that, the SEM micrographs of the samples at 45° inclination on the Žiri location after exposure show considerable inhomogeneities on the surfaces (Figure S5 in Supplementary Materials); they do not show, however, any measurable influences of the long-term exposure on the quality and morphology of the thin film surface itself.

However, the differences between the three surface materials (BioClean, float, TiZr) were not as pronounced. They all showed the same trend and none of the surfaces showed a significant deviation of the temporal evolution from the other two.

In addition to the position of the surfaces, the location of exposure also had a large influence on the visual appearance of the surfaces. The effect of Saharan sand at 480 days of exposure had the largest influence at the exposure site closer to the coastal region (93 m above sea level) and the lowest in the more hilly exposure site (478 m above sea level), which coincides with the amount of sand being deposited according to the meteorological models (Figure S4 in Supplementary Materials).

### 3.3. Anti-Fogging Effect

The differences between the surfaces were; however, more pronounced in laboratory testing of dewing times. Here the TiZr surface layer showed the longest time needed for large-enough droplets to form to scatter light and, hence, reduce the transparency of glass/coating (Figure 5). Not surprisingly, float glass formed visible water droplets first, followed by BioClean glass. All of the surfaces dewed faster when relative humidity was increased from 44% to 58% (Figure 5a). Interestingly, the time was also shorter if the samples were exposed in a vertical configuration as compared to the horizontal one (Figure 5b). This difference may seem unimportant, but, in a practical view, it means that windows that are installed in a more horizontal configuration will benefit more from the anti-fogging coating.

The effect of inclination angle can be explained considering the forces acting on the droplet in the two configurations. In the horizontal configuration, the gravity pulls the drop towards the surface and decreases the wetting contact angle. In addition, the same pulling force helps in adhesion on the water–glass interface and makes the formation of droplets harder than in the vertical position, hence the observed effect.

The explanation of the improvement in antifogging properties in the BioClean sample is elusive since the composition of the glass is not given by the producer; our characterization (Figure S6 in Supplementary Materials); however, has shown no presence of titania but rather suggests an organometallic coating. The antifogging effect of the TiZr coating, on the other hand, can be explained with the presence of titania phase, by the occurrence of oxygen defects upon UV-irradiation with the additional help of numerous Si–OH and Ti–OH surface groups already present on the particles of silica and titania, respectively. In this manner, measurements of the water contact angle (WCA, Figure 5c) of the three surfaces were made. Float glass showed a typical value of around 62°, while BioClean and the TiZr coating showed lower mean WCA values (i.e., around 22° and 15°, respectively). Zheng et al. [32] observed a similar effect in TiO_2_/VO_2_/TiO_2_ multilayer films and the higher hydrophilic nature of the films was correlated to higher anti-fogging effect, while the combined hydrophilic properties of SiO_2_ and TiO_2_, especially from SiO_2_ in the absence of UV irradiation, was found to be the reason for titania–silica coatings deposited on glass. [33] It must be noted that the measured hydrophilic nature of the TiZr coating probably is not the sole cause for the differences in dewing times, since the altered emissivity of the glass when coatings are applied probably has a large influence on droplet formation. This aspect of the materials is under current investigation.

**Figure 6:**
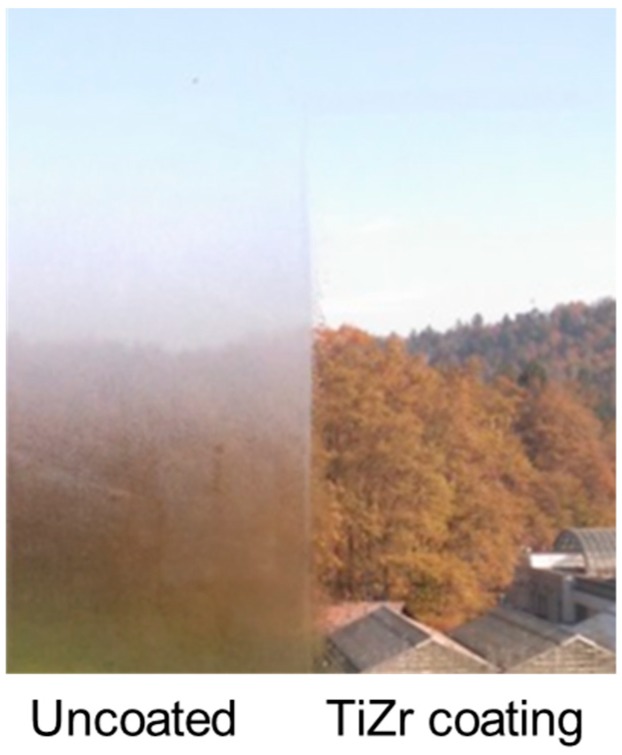
The anti-fogging effect of the TiZr coating on the office window. The picture was taken on 20 October 2017 after one month of exposure time.

The anti-fogging effect of the TiZr coating was well also demonstrated on the office window in Ljubljana (Figure 6), where we occasionally observed a remarkable difference between the uncoated and coated part of the glass exposed to the outdoor conditions. As a consequence of good insulation properties of the window, the humid outdoor air (a small stream is flowing adjacent to the building) condenses on the cold outer surface of the window, while such morning dew is not observed on the coated part of the window. As it is well known and expected from the highly hydrophilic nature of the titania–silica layer (like TiZr, see Figure 5c), its anti-fogging effect is well expressed also in rainy days; the observed effect is probably partially affected also by the low free-surface energy of such coatings, although it was not experimentally measured. The activity of the coating exposed to outdoor weather conditions did not deteriorate over the two-year period after its deposition. Additionally, the presence of SiO_2_ phase in the TiZr composite coatings is known to delay the crystallization of titania and produces smaller anatase particles, which, as shown in Figure 2a, are adequately crystalline. The suitable crystallinity of titania particles in these coatings is also a consequence of the presence of 10% of zirconium atoms in the TiO_2_; as we have shown before [15], the presence of Zr results in larger anatase particles at the same heat treatment temperature. Consequently this results in lower bandgap values also [15], hence more efficient use of the solar light in outdoor testing. Additionally, the presence of amorphous SiO_2_ phase improves the adhesion of the layer to the glass and abrasion resistance [34,35], which is clearly seen in long-term tests performed in this study (Figure 3c at 90° and Figure 6).

## 4. Conclusions

The outdoor performance of float, commercial self-cleaning, and sol-coated glass samples were systematically investigated over an outdoor exposure period of 20 months.

The effect of final installation orientation should not be overlooked when describing the possible applications of novel materials. We have shown that a surface in a vertical position behaves considerably different than the one positioned with a 45° angle.

The environmental conditions were shown to be an important factor, especially the amount of rain in combination with sun exposure; when these two factors were simultaneously present the self-cleaning effect was the most pronounced.

The self-cleaning property of either commercial BioClean glass or our TiZr coating material deposited on float glass was not found effective in real field test conditions, although previous laboratory tests showed good photocatalytic activity of TiZr thin films [15,23]. On the other hand, the anti-fogging effect of the TiZr coating was very well expressed in controlled laboratory conditions (measuring droplet formation time) as well as in the real outdoor environment.

## Supplementary Materials

The following are available online at https://www.mdpi.com/1996-1944/12/13/2196/s1, Figure S1: Field exposure setting-up (a), locations of the three set-ups (b) and images of the set-ups on different locations (c–e). Pictures were taken in January 2017; Figure S2: Measurements of light transmittance by means of lux meter; Figure S3: Initial measurements in the CIE L*a*b* system. The results of whiteboard were taken as a background for comparison of the other three samples throughout the exposure period; Figure S4: Position and concentration of the Saharan sand across Europe and Northern Africa in April 2018. Source: Environmental Agency of The Republic of Slovenia, https://twitter.com/meteoSI/status/984025061580517377?ref_src=twsrc%5Etfw; Figure S5: Scanning electron microscope (SEM) micrographs of surfaces after 1.5 years of exposure. The samples are from 45° inclination on the location of Žiri (ZI); Figure S6: SEM micrographs of the cross-cut BioClean glass with the profiles of elements across the cross-cut.
