# Supplementary material for: Field Test of Self-Cleaning Zr-Modified-TiO2-SiO2 Films on Glass with a Demonstration of Their Anti-Fogging Effect
Source: Materials (Basel). 2019 Jul 8;12(13):2196. doi: 10.3390/ma12132196 (PMC6651866; doi:10.3390/ma12132196)
Supplement: Supplementary file 1 [file materials-12-02196-s001.pdf]

materials

MDPI

Article

Field Test of Self-Cleaning Zr-Modified-TiO_2-SiO_2 Films on Glass with a Demonstration of Their Anti-Fogging Effect

Andraž Šuligoj [1,2,*], Olena Pliekhova [3], Nives Vodišek [3], Mohor Mihelčič [2], Angelja K. Surca [2], Roman Kunič [4], Barbara Šubic [5], Jernej Starman [5], Aleš Ugovšek [5] and Urška Lavrenčič Štangar [1,3]

1 Faculty of Chemistry and Chemical Technology, University of Ljubljana, Večna pot 113, SI-1000 Ljubljana, Slovenia; urska.lavrencic.stangar@fkkt.uni-lj.si

2 Department of Inorganic Chemistry and Technology, National Institute of Chemistry, Hajdrihova 19, SI-1000 Ljubljana, Slovenia; mohor.mihelcic@fs.uni-lj.si (M.M.); angelja.k.surca@ki.si (A.K.S.)

3 University of Nova Gorica, Vipavska 13, SI-5000 Nova Gorica, Slovenia; olena.pliekhova@ung.si (O.P.); nives.vodisek@gmail.com (N.V.)

4 Faculty of Civil and Geodetic Engineering, University of Ljubljana, Jamova 2, 1000 Ljubljana, Slovenia; roman.kunic@fgg.uni-lj.si

5 M-Sora d.d., Industrijska ul. 13, SI-4226 Žiri, Slovenia; barbara.subic@m-sora.si (B.S.); nejc.starman@m-sora.si (J.S.); ales.ugovsek@gzs.si (A.U.)

* Correspondence: andraz.suligoj@fkkt.uni-lj.si; Tel.: +386-1-479-8528

Supporting Information for the paper

1. Field exposure set-up

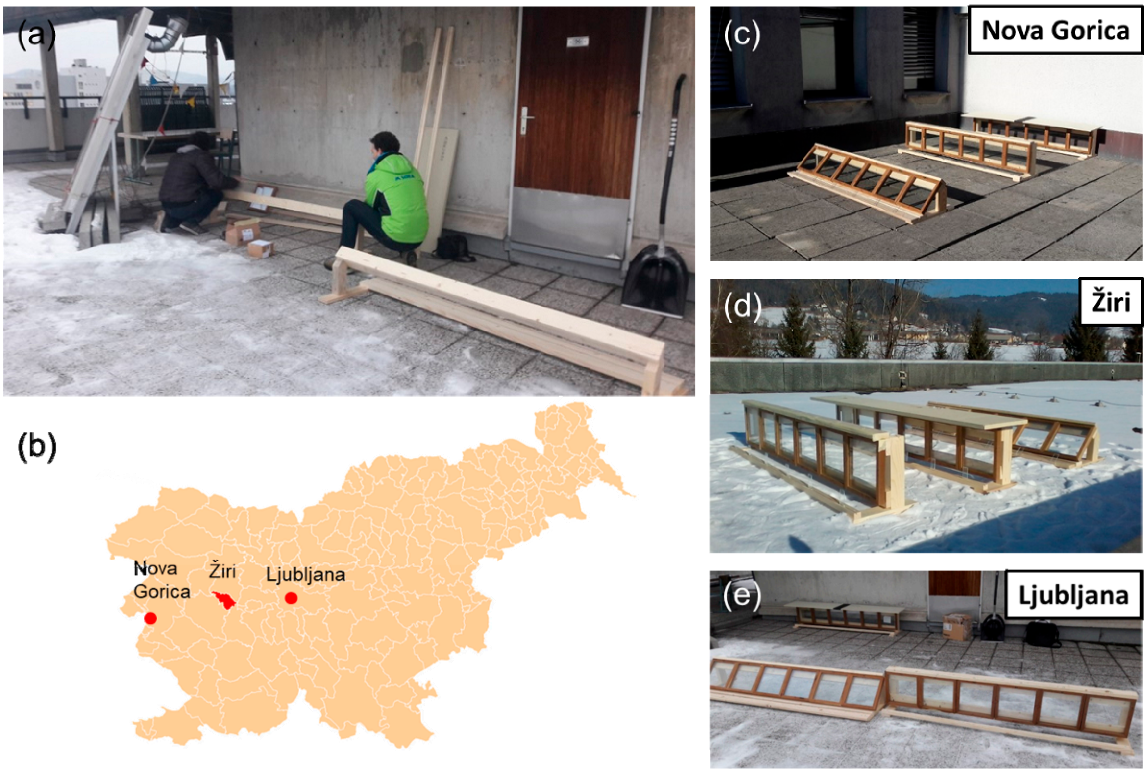

Figure S1. Field exposure setting-up (a), locations of the three set-ups (b) and images of the set-ups on different locations (c–e). Pictures were taken in January 2017.

2. In-field characterization techniques

The overview of transmittance measurements is shown in Figure S2. Each measurements was composed of three readings; one close to the left plank (as shown in the image), the second one in the middle of the glass and the third one closer to the right plank of the window frame.

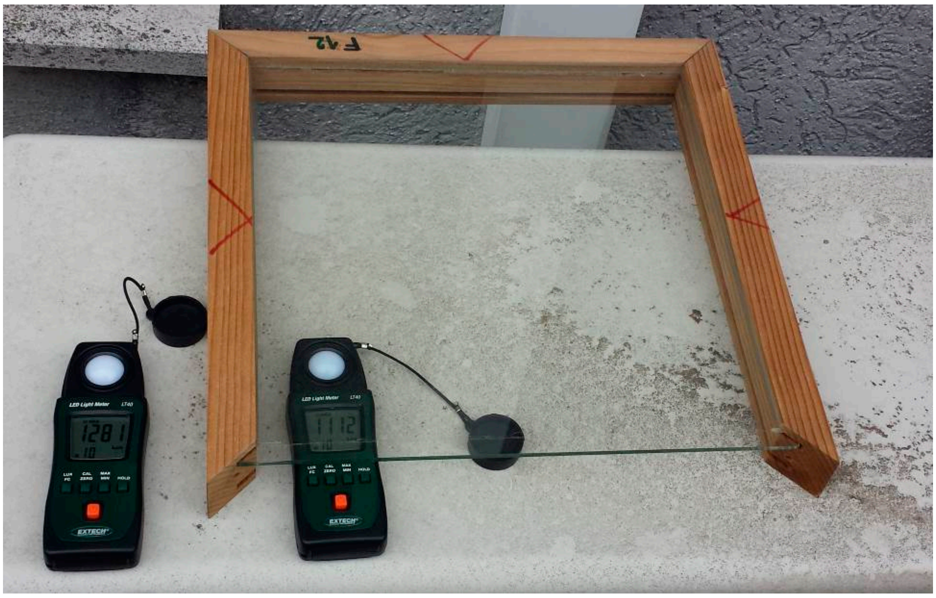

Figure S2. Measurements of light transmittance by means of Lux meter.

The initial measurements of color in the CIE L*a*b* system are discussed below. The measurements were made with the whiteboard as a background, which is essentially a wooden board with an acrylic paint layer. The results of this measurement served as a background for all other measurements of glass / coatings.

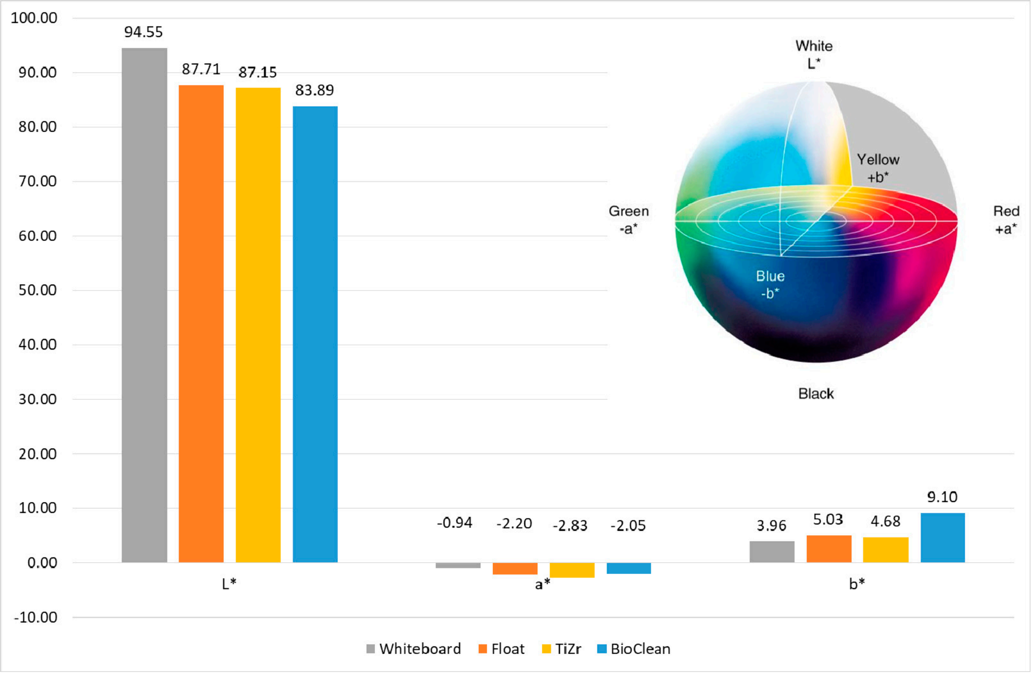

Figure S3. Initial measurements in the CIE L*a*b* system. The results of whiteboard were taken as a background for comparison of the other three samples throughout the exposure period.

3. Aerosol geospatial distribution

An overview of the position of the Saharan dust as it was present in April 2018 across Europe is shown in Figure S4.

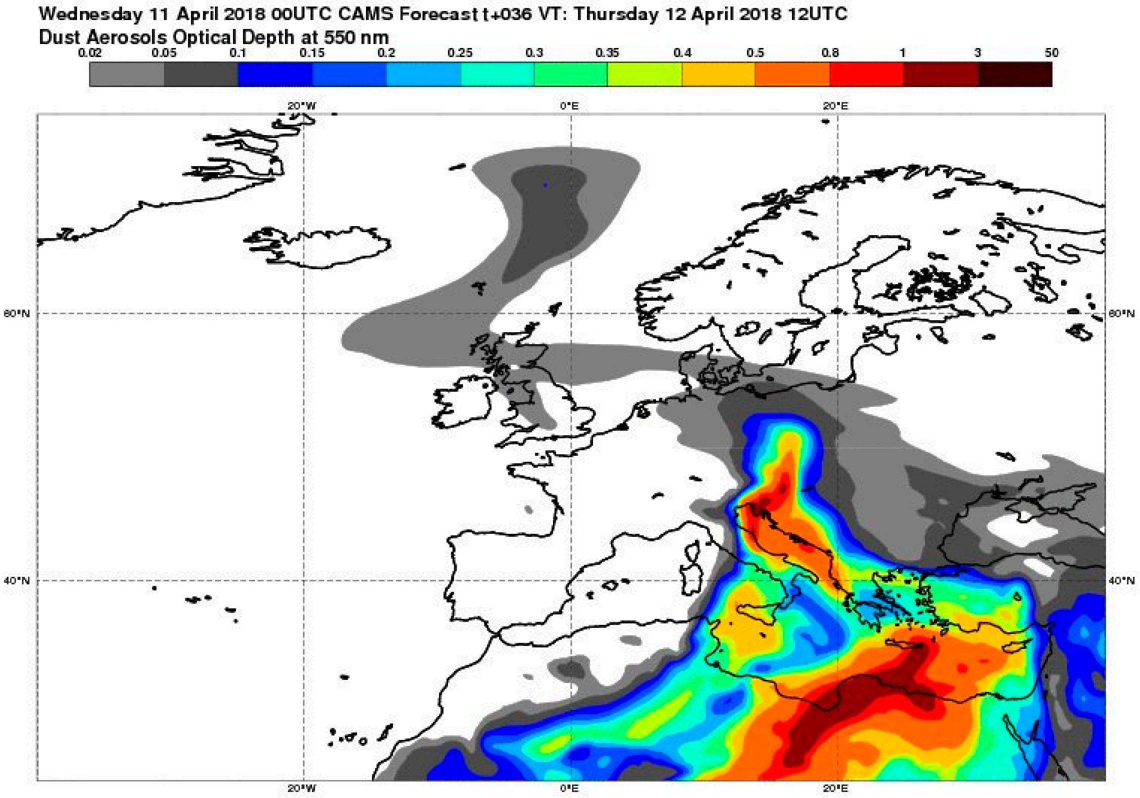

Figure S4. Position and concentration of the Saharan sand across Europe and Northern Africa in April 2018. Source: Environmental Agency of The Republic of Slovenia, https://twitter.com/meteoSI/status/984025061580517377?ref_src=twsrc%5Etfw.

4. Scanning electron microscope characterization

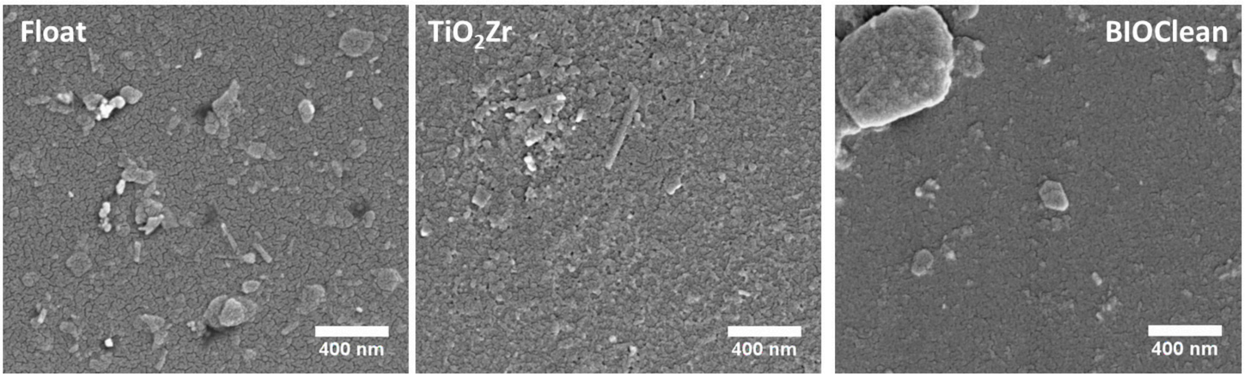

Figure S5. Scanning electron microscope (SEM) micrographs of surfaces after 1.5 years of exposure. The samples are from 45° inclination on the location of Žiri (ZI).

The cross cuts for SEM characterization were made by breaking the glass with clamps and a clean 90° angle piece of glass was investigated under the microscope. This characterization was done for BioClean sample alone.

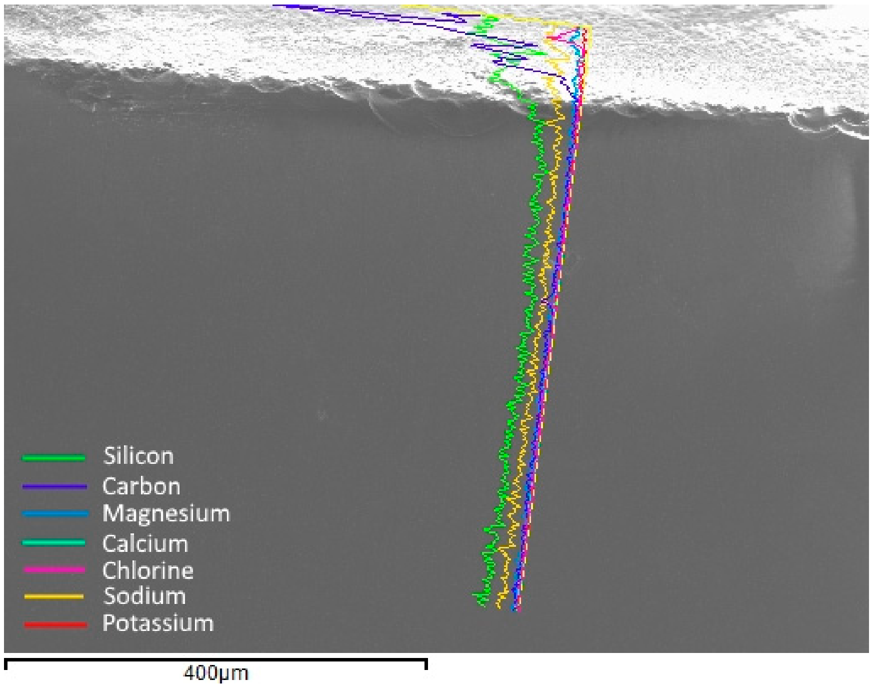

Figure S6. SEM micrographs of the cross-cut BioClean glass with the profiles of elements across the cross-cut.
